# Characterization of Arsenic-Resistant Endophytic Bacteria From Alfalfa and Chickpea Plants

**DOI:** 10.3389/fpls.2021.696750

**Published:** 2021-07-22

**Authors:** Hazhir Tashan, Behrouz Harighi, Jalal Rostamzadeh, Abdolbaset Azizi

**Affiliations:** ^1^Department of Life Science Engineering, Faculty of New Sciences and Technologies, University of Tehran, Tehran, Iran; ^2^Department of Plant Protection, Faculty of Agriculture, University of Kurdistan, Sanandaj, Iran; ^3^Department of Animal Sciences, Faculty of Agriculture, University of Kurdistan, Sanandaj, Iran

**Keywords:** alfalfa, arsenic resistance, chickpea, endophytic bacteria, plant growth-promoting

## Abstract

The present investigation was carried out to isolate arsenic (As)-resistant endophytic bacteria from the roots of alfalfa and chickpea plants grown in arsenic-contamination soil, characterize their As tolerance ability, plant growth-promoting characteristics, and their role to induce As resistance by the plant. A total of four root endophytic bacteria were isolated from plants grown in As-contaminated soil (160–260-mg As kg^−1^ of soil). These isolates were studied for plant growth-promoting (PGP) characteristics through siderophore, phosphate solubilization, nitrogen fixation, protease, and lipase production, and the presence of the arsenate reductase (*arsC*) gene. Based on *16S rDNA* sequence analysis, these isolates belong to the genera *Acinetobacter, Pseudomonas*, and *Rahnella*. All isolates were found As tolerant, of which one isolate, *Pseudomonas* sp. QNC1, showed the highest tolerance up to 350-mM concentration in the LB medium. All isolates exhibited phosphate solubilization activity. Siderophore production activity was shown by only *Pseudomonas* sp. QNC1, while nitrogen fixation activity was shown by only *Rahnella* sp. QNC2 isolate. *Acinetobacter* sp. QNA1, QNA2, and *Rahnella* sp. QNC2 exhibited lipase production, while only *Pseudomonas* sp. QNC1 was able to produce protease. The presence of the *arsC* gene was detected in all isolates. The effect of endophytic bacteria on biomass production of alfalfa and chickpea in five levels of arsenic concentrations (0-, 10-, 50-, 75-, and 100-mg kg^−1^ soil) was evaluated. The fresh and dry weights of roots of alfalfa and chickpea plants were decreased as the arsenic concentration of the soil was increased. Results indicate that the fresh and dry root weights of alfalfa and chickpea plants were significantly higher in endophytic bacteria-treated plants compared with non-treated plants. Inoculation of chickpea plants with *Pseudomonas* sp. QNC1 and *Rahnella* sp. QNC2 induced lower *NPR3* gene expression in chickpea roots grown in soil with the final concentration of 100-mg kg^−1^ sodium arsenate compared with the non-endophyte-treated control. The same results were obtained in *Acinetobacter* sp. QNA2-treated alfalfa plants grown in the soil plus 50-mg kg^−1^ sodium arsenate. These results demonstrated that arsenic-resistant endophytic bacteria are potential candidates to enhance plant-growth promotion in As contamination soils. Characterization of bacterial endophytes with plant growth potential can help us apply them to improve plant yield under stress conditions.

## Introduction

Plants are constantly affected by abiotic stress excreted by the environment. Heavy metals can reduce plant development and crop productivity when their concentration rises beyond supraoptimal values for plant normal functioning (Tiwari and Lata, 2018). As a consequence of industrialization during the past or from geogenic sources, the heavy metal concentration of soils has been increased worldwide (Adriano, 2001). Arsenic (As) is a heavy metal present in various soil and water ecosystem (Chowdhury et al., 1999; Abernathy et al., 2003). Arsenic contamination of natural environments is a major problem and has been reported in more than 20 countries (Jack et al., 2003). The contamination of as in groundwater used for irrigation not only affects plant growth but also accumulates in different plant tissues and contaminates the food chain (Tiwari and Lata, 2018). A high concentration of arsenic in water, plants, and soils has been reported from the south-eastern part of Kurdistan province, Iran (Mosaferi et al., 2003; Zandsalimi et al., 2011). The concentration of arsenic in these sites ranged from 100–300 mg kg^−1^ of soil. Heavy contamination of this trace element has adversely affected human health. Uptake and accumulation of arsenic in crops grown in polluted soils lead to poor growth and subsequent yield loss. Besides, arsenic can enter into edible parts of the plant, such as seeds and fruit, making them dangerous for consumers.

Excessive release of heavy metals into the environment results in plants to develop different mechanisms to cope with their negative effects (Franco-Franklin et al., 2021). A study of plant responses and tolerance revealed that several genes are induced under heavy metal stress. Heavy metals activate different signaling pathways in plants, such as calcium-dependent signaling, mitogen-activated protein kinase (MAPK) signaling, reactive oxygen species (ROS) signaling, and phytohormonal response (Dutta et al., 2018).

Plants are naturally associated with diverse microorganisms in various ways. Among them, endophytic bacteria can be defined as those bacteria that colonize the internal tissue of the plant, showing no external sign of infection or negative effect on their host (Schulz and Boyle, 2006). Endophytic bacteria can promote plant growth by several mechanisms that include indole acetic acid (IAA) synthesis, phosphate solubilization activity, production of siderophore under Fe-limiting conditions, improving mineral nutrient uptake by plants, and nitrogen fixation activity (Ryan et al., 2008). Endophytic bacteria can promote the growth of the plant hosts, even under abiotic stress conditions (Franco-Franklin et al., 2021). Several reports also indicate the induction of genes in plants that are central to heavy metal stress signaling in the presence of beneficial bacteria, such as *Pseudomonas* and *Bacillus* (Tiwari and Lata, 2018).

It is well-known that bacteria isolated from polluted habitats are tolerant to higher levels of heavy-metal concentration (Sessitsch and Puschenreiter, 2008). Arsenic-tolerant bacteria were described more than a century ago (Green, 1918; Green and Kestell, 1920). They play an important role in the transformation of arsenic, thus affecting its bioavailability and toxicity (Oremland and Stolz, 2005). Recently, Satapute et al. (2019) have reported *Pseudomonas taiwanensis* strain resistant to arsenic. Genes responsible for arsenic tolerance have been identified in many bacteria and organized in the *ars* cluster (Ben Fekih et al., 2018). Recent research has shown that endophytic bacteria play important roles in plant response to abiotic stresses, helping the plant host to adapt to the ecological niche occupied (Sim et al., 2019). Indeed, some of the endophytic bacteria could alleviate plant hosts from heavy metals toxicity (Román-Ponce et al., 2018). Several mechanisms in the arsenic-resistant bacteria have been characterized that enable them to affect the arsenic mobility, solubility, and toxicity in the contaminated environments (Gadd, 2010). Several studies have shown that several endophytic bacteria are suitable candidates for reducing the arsenic stress to the host plants (Nie et al., 2002; Wang et al., 2011; Babu et al., 2013; Mesa et al., 2017).

Chickpea (*Cicer arietinum* L.) and alfalfa (*Medicago sativa* L.) are rich sources of seed proteins and primarily grown for use in animal feed, respectively, and consumed worldwide. In Iran, chickpea and alfalfa plants, in ~450 and 500,000 hectares of land, are planted. Total production is estimated at over 200,000 and 4 million tons, respectively, and they are among the top five most important crops growing in various parts of Iran (Anonymous, 2019). Both plants are also being grown in arsenic-contaminated areas in Iran. Despite its economical importance, not much study exists on the negative effects of As on these plants. Previous study reported As toxicity on chickpea plants grown in As-contaminated situations (Malik et al., 2011). The previous studies on alfalfa and chickpea plants grown in arsenic-contaminated conditions showed this heavy metal to be reduced plant biomass production (Saadati et al., 2017). Srivastava and Singh (2014) reported arsenic-tolerant chickpea growth-promoting bacteria isolated from As-contaminated sites. Although the use of endophytic bacteria to promote the growth of plant hosts had been reported, their influence on increased resistant to high concentration of As is still not well-understood. Therefore, this study aimed to identify endophytic bacteria resistance to the high concentrations of arsenic isolated from contaminated areas, examined the effects of arsenic on biomass production of alfalfa and chickpea, inoculated with these strains, and evaluated the effects of these bacteria on the induction of systemic resistance in plants grown in an As-polluted soil.

## Materials and Methods

### Isolation and Identification of Endophytic Bacteria

The sampling sites are located in Neyband in the Kurdistan province. Chickpea (*Cicer arietinum* var. Kabuli ILC482) and alfalfa (*Medicago sativa* var. Hamedani) plants were collected from arable land sampling site 1 (35° 12′ N, 48° 04′ E) and sampling site 2 (35° 12′ N, 48° 7′ E), with the altitude of about 2,040 and 2,021 m, respectively, during June–July 2019. The two sampling sites have a distance of about 4.5 km. The total arsenic concentrations of about 260- and 160-mg kg^−1^ soil have been detected in the sampling sites 1 and 2, respectively. In total, eight samples from each chickpea and alfalfa plants were collected during the vegetative growth stage.

For isolation of endophytic bacteria, 1 g of roots was washed with tap water, surface sterilized with 0.5% sodium hypochlorite solution for 2–3 min, and rinsed three times in sterile distilled water. Afterward, they were crushed in 5-ml sterile distilled water and cultured on yeast extract mannitol agar (YMA) or nutrient agar (NA) media. To confirm that the disinfection process was successful, the aliquots of the sterile distilled water used in the final rinse were cultured on an NA medium. Plates were incubated at 26–28°C for 14 h, and single colonies with different morphologies were purified onto the same medium. Bacteria were grown in the Luria-Bertani (LB) medium at 26–28°C for 24–48 h; sterile glycerol was added to a final concentration of 20% and stored at −70°C.

Genomic DNA of endophytic bacteria was extracted from each isolate. Overnight-grown culture of bacteria in 5 ml of LB medium was treated by adding SDS/lysozyme, followed by three-time DNA extraction with phenol/chloroform/isoamyl alcohol (25/24/1 volume) and once with chloroform/isoamyl alcohol (24/1). DNA was precipitated with.1-volume 3-M sodium acetate (pH: 4.8) and two volumes of absolute ethanol overnight at −20°C. Finally, the suspension was centrifuged at 14,000 rpm for 5 min; DNA was washed with 70% ethanol, dried, and resuspended in 50 μl of TE (Tris-Cl 0.1M, EDTA 0.01M, pH:8) buffer. Through PCR reactions, a DNA fragment corresponding to *16S rRNA* gene was amplified by using two universal primers, fD2 (5′-AGA GTT TGA TCA TGG CTC AG-3′, position 8-27) and rP1 [5′-ACG GTT ACC TTG TTA CGA CTT-3′, position 1,512-1,492 (*Escherichia coli*)], following the method described by Weisburg et al. (1991). The purified amplicons were sequenced, using an ABI3730XL DNA sequencer (Applied Biosystems, Foster City, CA, USA). The *16S rRNA* sequences were manually edited with BioEdit Sequence Alignment Editor 7.0.9.0 software (Hall, 2011). The phylogenetic tree was constructed by the neighbor-joining method with Kimura's two-parameter calculation model in MEGA version 6.0 (Tamura et al., 2013). The reliability of the phylogenetic analysis was evaluated by bootstrap analysis with 1,000 replicates, using the same program. The sequences obtained were deposited in the GenBank under accession numbers MN658698 to MN658701.

### Characterization of Isolated Endophytic Bacteria

The presence of the *arsC* gene was targeted to detect arsenic resistance in isolated endophytes. PCR was performed, using the degenerate primers arsCF (5′-ATGACCGTCACCATHTAYCAYAAYC-3′) and arsCR (5′- ACGACCGCCTCGCCRTCYTCYTT-3′) located 1–25 and 393–415 bp, respectively, under the following conditions: 1 cycle at 95°C for 3 min; 35 cycles at 95°C for 1 min, 48°C for 1 min; and 72°C for 45 s; and a final extension cycle at 72°C for 10 min (Sa-Pereira et al., 2007). The PCR products were sequenced as described above. The sequences were used for a Blast search in the NCBI GenBank database to verify the gene identity. The sequences obtained were deposited in the GenBank under accession numbers MN782313 and MN782314.

Phosphate solubilization activity was assayed on Pikovskaya agar medium according to Naik et al. (2008). Siderophore production was measured on chrome azurol-S (CAS) medium, following the method previously described (Schwyn and Neilands, 1987). The ability of isolates to fix nitrogen on nitrogen-free NFb medium was assessed, using methods previously described by Döbereiner et al. (1995).

The production of protease and lipase was screened for each bacterial isolate. Protease production was determined according to the method previously described (Sgroy et al., 2009), with some modifications. Plates containing skim milk agar (SMA) medium (g^−1^ of distilled water: a pancreatic digest of casein, 5; yeast extract, 2.5; glucose, 1; 7% skim milk and agar, 15) were inoculated with 10 μl of freshly prepared bacterial isolates, incubated at 28°C for up to 4 days and observed for the formation of the halo zone around the colonies. Lipase production by endophytic bacteria was tested based on the method described by Sierra (1957). A nutrient agar medium was prepared, and tween 80 was added to a final concentration of about 1%. The bacterial suspension was spot inoculated on media; the plates were incubated at 26–28°C and observed for the production of an opaque halo around the colonies.

To investigate the arsenic tolerance property, bacterial strains were grown in the LB liquid medium at 26–28°C for 24 h. Cultures were centrifuged (5 min, 7,000 rpm), and the pellet was suspended in sterile-distilled water to a concentration of ~1 × 10^7^ CFU ml^−1^ (OD_600nm_ = 0.1). About 10 μl of suspension was inoculated into culture tubes of 10-ml LB media, containing sodium arsenate (Na_2_HAsO_4_.7H_2_O) at final concentrations of 150, 200, 250, 300, 350, and 400 mM. Inoculated media were incubated at 26–28°C with shaking at 150 rpm. The minimum inhibitory concentration (MIC) at which bacterial strains failed to grow was monitored by measuring OD_600__nm_ after 72 h. Inoculated media without sodium arsenate was used as a control. Assays were conducted with three replications.

### Effect of Endophytic Bacteria on Biomass Production

Pots containing steam-sterilized soil (As: 62.933 ppb; Silica: 28.44%; Clay: 14.44%; Sand: 57.12%; EC: 2.7 ms cm^−1^; pH: 7.24) plus 10-, 50-, 75-, 100-mg kg^−1^ soil of sodium arsenate or without As (control) were prepared and incubated for 4 weeks to allow the As to equilibrate. After that, seeds of alfalfa (var. Hamedani) and chickpea (Kabuli ILC482) were sterilized and potted. After adding 5 ml from 1 × 10^10^ CFU ml^−1^ of each bacterial strain, pots were placed in the greenhouse (28–30°C; 16:8 day/night photoperiod) and watered every 2 days. Eight weeks after inoculation, the plants were harvested, the roots with nodules excised, and the number of nodules per plant was counted. Fresh roots of each plant were weighted and rinsed three times in distilled water before being dried for 48 h at 70°C, and dry roots were weighted. The experiments were carried out with three replications, each pot containing five plants, and the results are the mean of at least three harvested plant samples.

### Plant Colonization

Ten days after inoculation of alfalfa and chickpea plants with endophytic bacteria, 200-mg samples of roots, stems, and leaves were selected, surface sterilized with 0.8% sodium hypochlorite for 5 min, and washed several times with sterile water. Plant tissues were then homogenized aseptically with a pestle and mortar in 1-ml distilled water. Aliquots of 30 μl were spread onto nutrient agar media, incubated at 26–28°C, and, after 48 h, the colonies were counted as CFU g^−1^ of plant tissues. All the experiments were repeated three times with three replicates, using control healthy plants inoculated with a PBS buffer.

$$\begin{array}{l} \text{Fold change due to treatment}=2_{\text{T}}^{-\Delta \Delta \text{C}}\\ \;=2^{-\left[\text{CTof the target gene }-\text{CT of the reference gene}\right]\text{ sample A}-\left[\text{CT of the target gene }-\text{CT of the reference gene}\right]\text{ sample B}.} \end{array}$$

### *NPR3* Gene Expression Analysis

Roots of endophytic bacteria-treated plants grown in soil containing 50 and/or 100 mg kg^−1^ of sodium arsenate (sample A) and untreated plants grown in soil without sodium arsenate (sample B) were collected after 10 days, grounded in liquid nitrogen, and stored in a sterile microtube at −70°C until used. For total RNA extraction, 50 mg of frozen powdered tissues were homogenized in 1 ml of TRIzol reagent and incubated for 5 min at room temperature. Then, 0.2 ml of chloroform was added to the sample, vortexed vigorously for 15 s, and incubated at room temperature for 2–3 min. Samples were centrifuged (12,000 × g, 10 min, 4°C), and the upper aqueous phase was transferred into a fresh tube. For RNA precipitation, the aqueous phase was mixed with an equal volume of isopropyl alcohol; the sample was incubated at 15 to 30°C for 10 min and centrifuged (12,000 × g, 10 min, 4°C). The supernatant was removed completely, an RNA pellet was washed with 70% ethanol and centrifuged at 7,000 × g for 10 min at 4°C. The above washing procedure was repeated one time and RNA pellet was air-dried for 5–10 min. RNA was dissolved in 50-μl DEPC-treated water and stored at −70°C until used. The concentration and the purity of RNA were determined by measuring the ratio of OD at 260/280 nm, using Multidrop^TM^ Pico 1 Digital Dispenser (Thermo Fisher Scientific, USA).

cDNA was amplified from 200 ng of total RNA, using the cDNA synthesis Kit (Yekta Tajhiz Azma, Iran) according to the instructions of the manufacturer. The expression of the targeted gene, including the *NPR3*, was checked in roots 10 days after treated by endophytic bacteria. Real-time PCR (RT-PCR) was performed with SYBR Green qPCR Master Mix (Yekta Tajhiz Azma, Iran), using an AB Applied Biosystem StepOne thermal cycler. PCR reactions were carried out in a 15-μl final volume, containing 5 μl of SYBR Green Master Mix, 10 μM forward and reverse primers (Table 1), and 1 μl of 10-fold diluted cDNA. Cycling conditions were 1 min at 95°C, followed by 40 cycles of 20 s at 95°C; 30 s, 58°C; and 30 s at 72°C. Traditional reference gene *EF1*α, with stable expression, was used as internal controls (a housekeeping gene) for normalization. Several studies have shown the stability of the EF1α gene for RT-PCR in alfalfa and chickpea plants (Garg et al., 2010; Wang et al., 2015). Before RT-PCR, the specificity of each primer was checked, using heat dissociation at 60°C. Relative gene expression was calculated with the following formula (Schmittgen and Livak, 2008):

**Table 1:** Primer sequences used for RT_PCR analysis of a defense-related gene.

**Primer**	**Sequence (5^**′**^->3^**′**^)**	**Length**	**Tm**	**GC%**	**Self complementarity**	**Self 3^**′**^ complementarity**
*EF1α* Forward	TCTGTTGTTGTAACAAGATGGATGC	25	60.05	40.00	8.00	2.00
*EF1α* Reverse	AAGGTTGGTGGACCTCTCAATC	22	59.96	50.00	5.00	5.00
*NPR3* Forward	GTACCTTGAGAACAGAGTGGCA	22	59.96	50.00	4.00	3.00
*NPR3* Reverse	GAAAGACCAGCAAACTCGGATG	22	59.84	50.00	3.00	1.00

CT values were the means of three biological replications and three technical replications.

### Statistical Analysis

The experiments were carried out with a factorial arrangement in a completely randomized design with three replications, two levels of inoculation, and five levels of sodium arsenate concentration (0-, 10-, 50-, 75-, and 100-mg kg^−1^ soil). Data analysis was performed, using the SAS software program, and a comparison of means was done with Fisher's least significant difference (LSD) test at a 5% level. Normality of data was verified, using the Shapiro–Wilk test. The graphs were plotted with Excel software.

## Results

### Identification of Endophytic Bacteria

**Figure 1:**
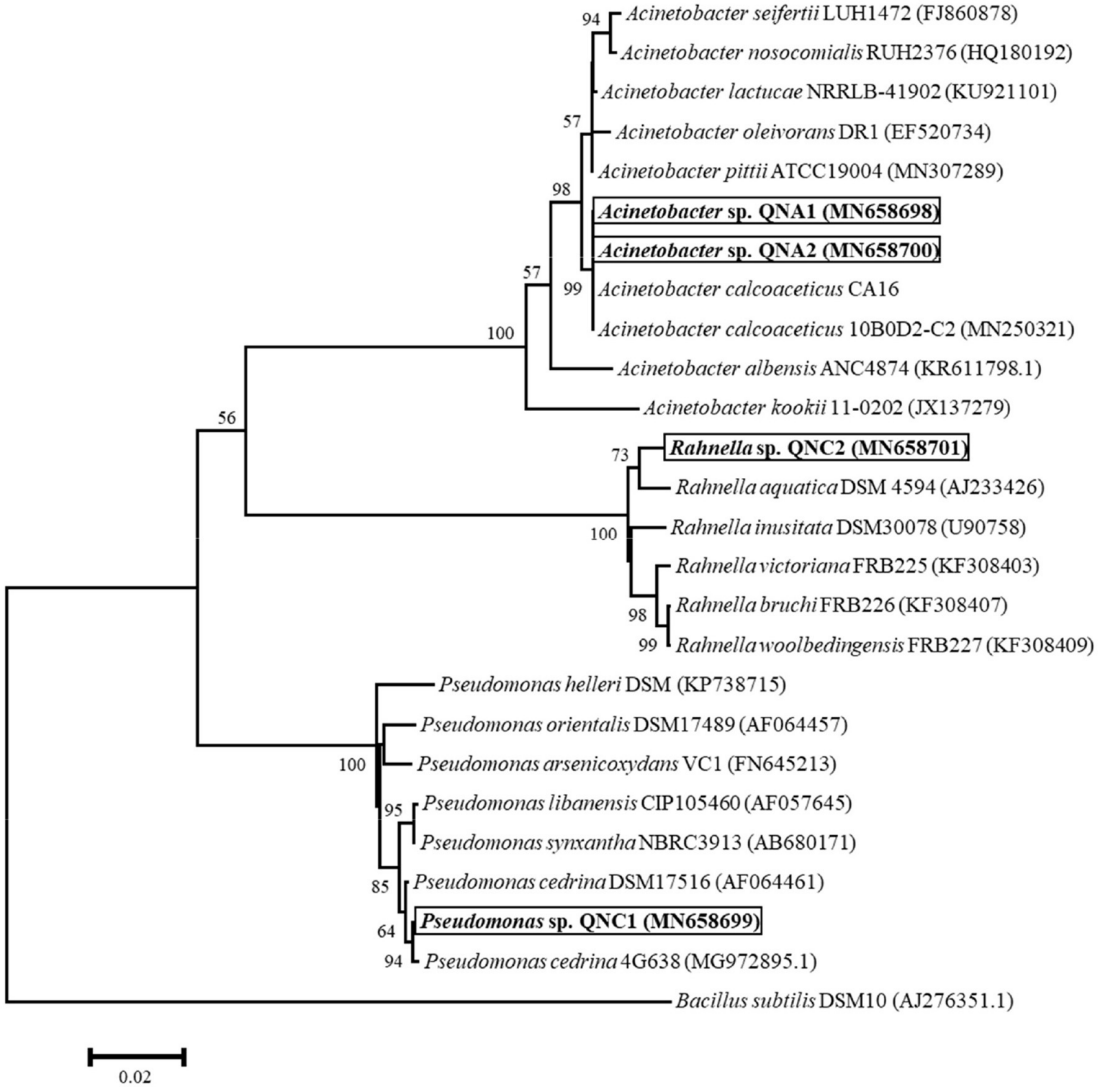
The phylogenetic tree of partial *16s rRNA* gene sequences, showing the position of *Pseudomonas* sp. QNC1, *Rahnella* sp. QNC2, and *Acinetobacter* sp. QNA1 and QNA2 strains in addition to taxonomically closest reference strains. The analysis was conducted by the neighbor-joining method with Kimura's two-parameter calculation model in MEGA version 6.0. The scale bar represents the number of substitutions per site. Numbers at branching points indicate bootstrap value derived from 1,000 replicates.

A total of four cultivable endophytic bacteria were isolated from the roots, including two from alfalfa (QNA1 and QNA2) and two from chickpea (QNC1 and QNC2). Based on the *16S rRNA* gene sequencing results, a phylogenetic tree was constructed, showing the position of strains within the type strains of the species of the genera *Pseudomonas, Acinetobacter*, and *Rahnella* (Figure 1). The phylogenetic analysis illustrated that two strains, namely QNA1 (MN658698) and QNA2 (MN658700) were grouped among species of the genus *Acinetobacter* and showing the closest 100% similarity with *A. calcoaceticus* CA16, a bacterium isolated from Canadian soil with bioremediation potential (Ho et al., 2020). QNA1 and QNA2 strains showed 100% similarity; therefore, QNA2 was selected for further analysis. Strain QNC1 (MN658699) formed a subclade and showing 99% similarity with *Pseudomonas cedrina* DSM17516 within the genus *Pseudomonas*. Also, QNC2 (MN658701) formed a subclade and showed the closest 99% similarity with *Rahnella aquatica* DSM4594.

### Molecular Characterization of the Arsenic Resistant Isolate

The arsenic resistance gene, namely, *arsC*, reported for the biosynthesis of arsenate reductase in bacteria was targeted to detect the isolated endophytic bacteria. PCR amplification, using specific primers, results in the production of an expected DNA fragment of about 400 bp in QNA2, QNC1, and, QNC2 strains. The resulting PCR product sequences obtained from QNA2 and QNC2 showed the closest homology with arsenate reductase gene sequences previously reported in *Acinetobacter calcoaceticus* str. 2117 and *Rahnella aquatilis* HX2, respectively.

### Plant Growth-Promoting and Enzymatic Characteristics

Endophytic bacterial strains were assayed for several traits that are important for plant growth, and the results are summarized in Table 2. *Acinetobacter* sp. QNA2, *Rahnella* sp. QNC2 and *Pseudomonas* sp. QNC1 strains were able to solubilize phosphate. Among the endophytic bacteria tested, siderophore production and nitrogen fixation activity were found in *Pseudomonas* sp. QNC1 and *Rahnella* sp. QNC2, respectively. The strain *Pseudomonas* sp. QNC1 showed positive for protease production, while *Acinetobacter* sp. QNA2 and *Rahnella* sp. QNC2 were able to produce lipase.

**Table 2:** Plant growth potential properties of endophytic bacterial strains isolated from alfalfa and chickpea plants.

**Strain**	**Phosphate solubilization**	**Siderophore production**	**Nitrogen fixation activity**	**Protease production**	**Lipase production**
QNA2	+	–	–	–	+
QNC1	+	+	–	+	–
QNC2	+	–	+	–	+

### Arsenic Tolerance

**Figure 2:**
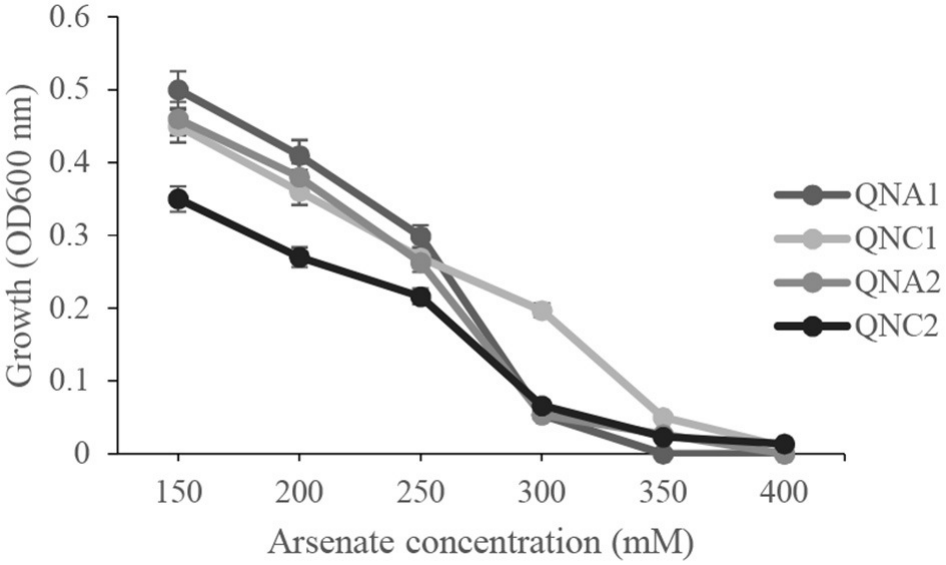
Effect of different concentrations of sodium arsenate on the growth of *Pseudomonas* sp. QNC1, *Rahnella* sp. QNC2, *Acinetobacter* sp. QNA1 and *Acinetobacter* sp. QNA2.

The isolated bacterial endophytes were further tested for arsenic tolerance by determining their growth in liquid media, containing 150–400 mM sodium arsenate. The minimal inhibitory concentration of arsenate against *Rahnella* sp. QNC2 and *Acinetobacter* sp. QNA1 and QNA2 was 300 mM, while only strain *Pseudomonas* sp. QNC1 showed tolerance to 350-mM sodium arsenate concentration (Figure 2).

### Plant Biomass Production

**Figure 3:**
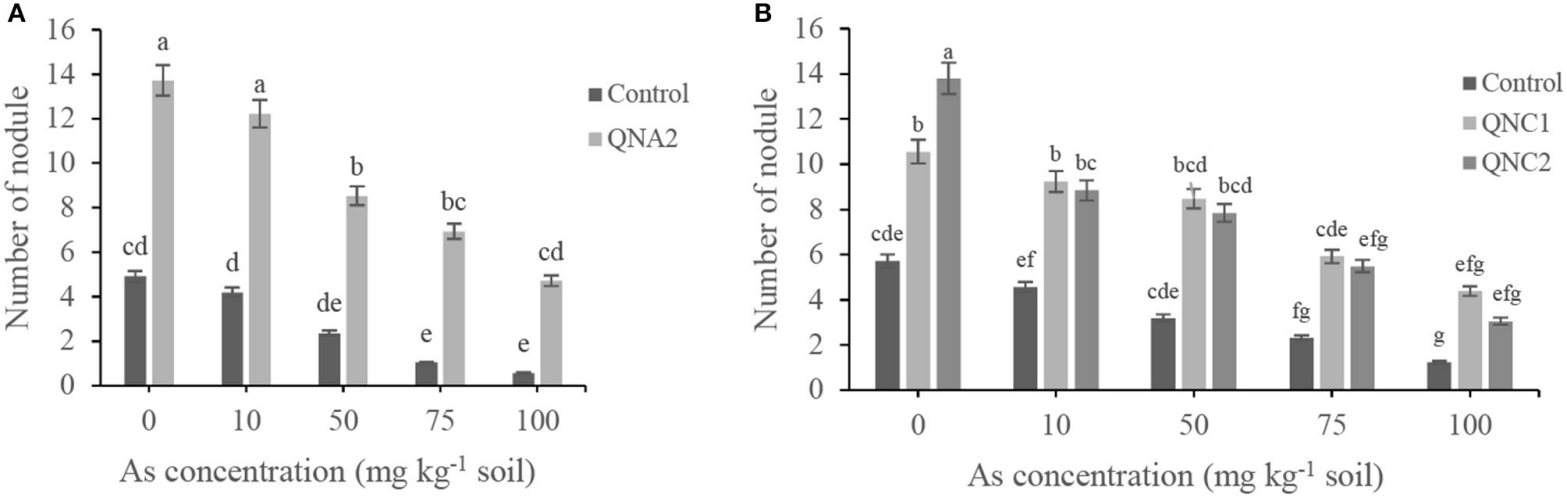
Effect of different sodium arsenate concentrations on root nodule production of **(A)** alfalfa plants treated with *Acinetobacter* sp. QNA2, **(B)** chickpea plants treated with *Pseudomonas* sp. QNC1 and *Rahnella* sp. QNC2. Data are the mean of three individual repetitions. Error bars indicate a standard error, and different letters above error bars represent significant differences based on a least significant difference (LSD) test (*P* = 0.05).

Endophytic bacterial strains were screened for their plant growth-promoting attributes in soil supplemented with sodium arsenate. The number of nodules and root biomass of treated plants with endophytic bacteria was increased compared with non-treated plants. The number of nodules of alfalfa and chickpea plants was decreased as the As concentration was increased. For all of the traits, alfalfa plants treated with QNA2 showed significantly increased 2.8-, 2.9-, 3.6-, 6.9-, and 8.2-fold nodule numbers in plants grown in the presence of As (0-, 10-, 50-, 75-, and 100-mg kg^−1^ soil), respectively, than non-treated plants (Figure 3A). Chickpea plants treated with QNC1 showed significantly increased 1.8-, 2.0-, 2.6-, 2.5-, and 2.4-fold nodule numbers in plants grown in the presence of As (0-, 10-, 50-, 75-, and 100-mg kg^−1^ soil), respectively, than non-treated plants. In comparison, the endophytic strains QNC2 significantly increased nodule numbers by 2.4-, 1.9-, 2.4-, 2.3-, and 2.4-fold in plants grown in the presence of As (0-, 10-, 50-, 75-, and 100-mg kg^−1^ soil), respectively, than non-treated plants. However, the number of nodules of plants treated with QNC1 did not significantly differ from that of those treated with QNC2 (Figure 3B).

**Figure 4:**
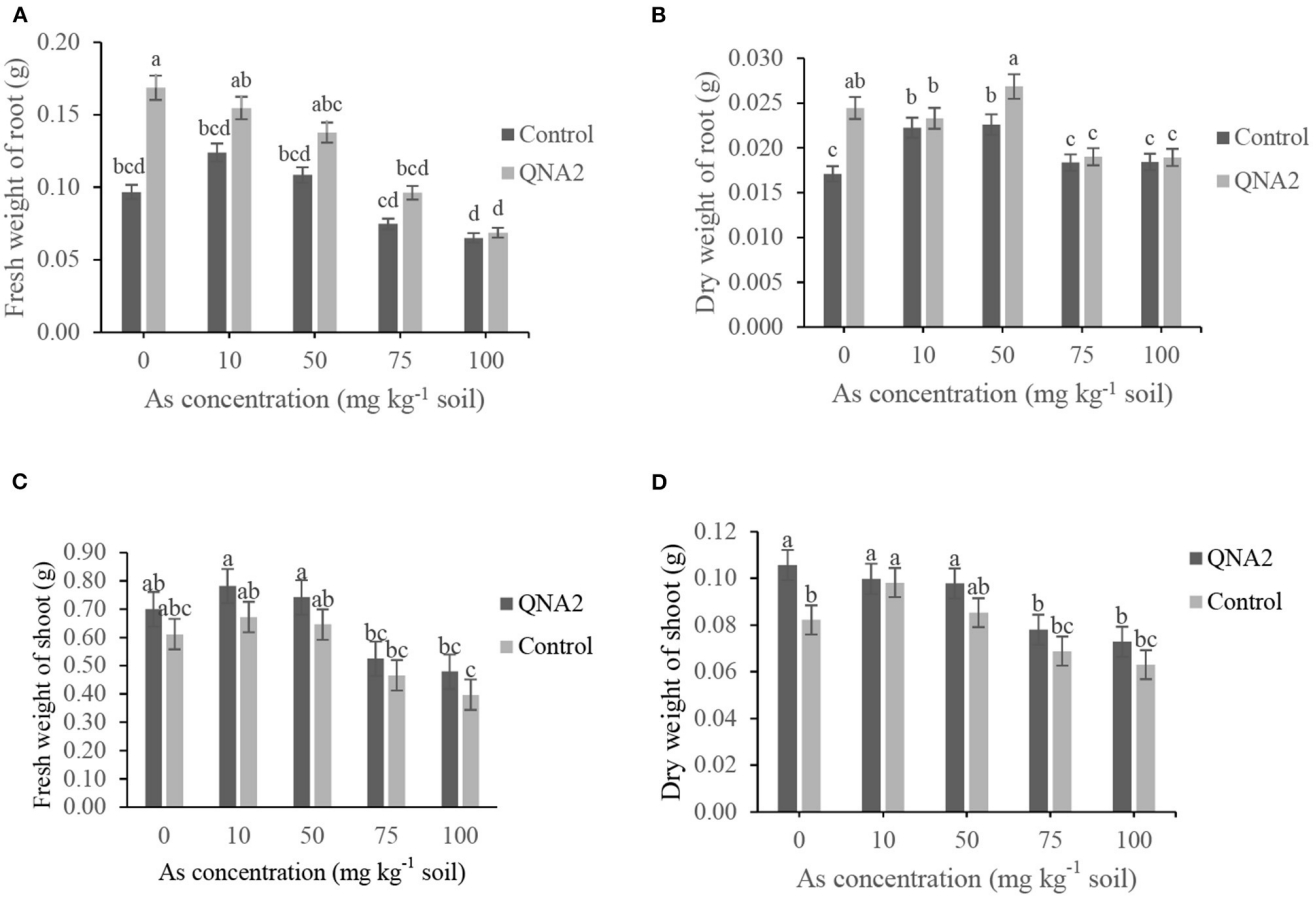
Effect of different sodium arsenate concentrations on **(A)** the fresh weights of roots, **(B)** dry weights of roots **(C)** fresh weights shoot and **(D)** dry weights shoot biomass of alfalfa plants treated with *Acinetobacter* sp. QNA2. Data are the mean of three individual repetitions. Error bars indicate a standard error, and different letters above error bars represent significant differences based on a least significant difference (LSD) test (P =0.05).

The fresh and dry weights of alfalfa roots were decreased as the As concentration of the soil was increased, both in the treated plants with QNA2 strain and the non-treated control (Figures 4A,B). In comparison with non-treated alfalfa, the endophytic strain QNA2 increased the root fresh weights by 74, 24, 26, 28, and 5% and root dry weights by 42, 4, 18, 3, and 2% in plants grown in the presence of As (0-, 10-, 50-, 75-, and 100-mg kg^−1^ soil), respectively. In the presence of As (50-mg kg^−1^ soil), significantly higher root biomass was observed in the plants treated with QNA2 than in the control. Similarly, the fresh and dry weights of alfalfa shoots were decreased as the As concentration of the soil was increased, both in the treated plants with QNA2 strain and the non-treated control (Figures 4C,D). The endophytic strain QNA2 increased the shoot fresh weights by 14, 16, 14, 13, 20% and shoot dry weights by 28, 1, 15, 13, and 14% in plants grown in the presence of As (0-, 10-, 50-, 75-, and 100-mg kg^−1^ soil), respectively, compared with non-treated alfalfa plants.

**Figure 5:**
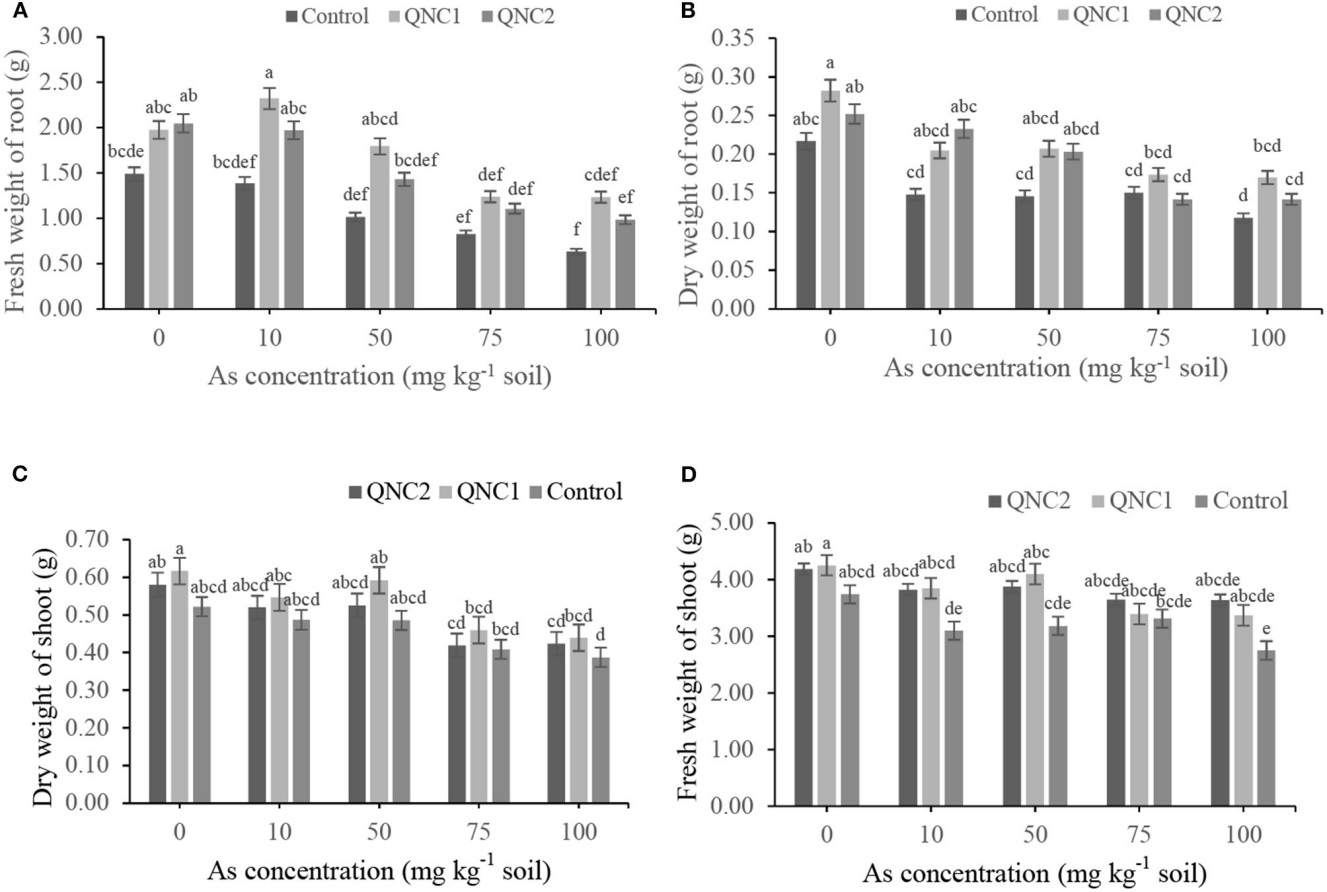
Effect of different sodium arsenate concentrations on **(A)** the fresh weights of roots, **(B)** dry weights of roots **(C)** fresh weights shoot and **(D)** dry weights shoot biomass of chickpea plants treated with *Pseudomonas* sp. QNC1 and *Rahnella* sp. QNC2. Data are the mean of three individual repetitions. Error bars indicate a standard error, and different letters above error bars represent significant differences based on a least significant difference (LSD) test (P =0.05).

The fresh and dry weights of chickpea roots were decreased as the As concentration of the soil was increased, both in the treated plants with QNC1 and QNC2 strains and the non-treated control (Figures 5A,B). In comparison with non-treated chickpea, the endophytic strain QNC1 increased the root fresh weights by 32, 67, 77, 50, 94% and root dry weights by 30, 38, 42, 15, and 44% in plants grown in the presence of As (0-, 10-, 50-, 75-, and 100-mg kg^−1^ soil), respectively. Chickpea plants treated with QNC2 increased the root fresh weights by 37, 42, 41, 34, 55% and root dry weight by 16, 57, 39, and 20% in plants grown in the presence of As (0, 10, 50, 75, and 100-mg kg^−1^ soil), respectively, than the non-treated plants. Although the plants treated with QNC1 and QNC2 always presented significantly higher fresh and dry weight of roots compared with control, no significant differences occurred between the two strains. Compared with non-treated chickpea, QNC1 strain significantly increased the shoot fresh weights by 13, 24, 26, 2, 22%, and shoot dry weights by 18, 12, 21, 12, and 9% in plants grown in the presence of As (0-, 10-, 50-, 75-, and 100-mg kg^−1^ soil), respectively. Chickpea plants treated with QNC2 increased the shoot fresh weights by 11, 23, 21, 10, and 32% and shoot dry weight by 11, 6, 8, 2, and 9% in plants grown in the presence of As (0-, 10-, 50-, 75-, and 100-mg kg^−1^ soil), respectively. In the majority of treatments, QNC1 and QNC2 strains showed increasing effects on fresh and dry shoot weights compared with control; no significant differences occurred between the two strains.

### Colonization of Plants by Endophytic Bacteria

Ten days after treatment of alfalfa and chickpea plants with bacterial endophytes, shoot and root colonization was assessed quantitatively (CFU g^−1^ of plant tissues). Colonies similar to QNA2, QNC1, and QNC2 strains were recovered from both the surface-sterilized shoots and roots of plants suggesting that these bacteria were able to colonize and/or spread throughout the internal tissues of plants. No bacteria were recovered from the control plants. In all treatments, the number of bacteria was decreased as the As concentration of the soil was increased. For all of the traits, the number of bacteria recovered from the roots was higher than that recovered from the shoots (Table 3).

**Table 3:** The population of *Acinetobacter* sp. QNA2 in shoots and roots of alfalfa plants, *Pseudomonas* sp. QNC1 and *Rahnella* sp. QNC2 in chickpea plants grown in soils, containing 0-, 10-, 50-, 75- and 100-mg kg^−1^ sodium arsenate 10 days after treatment.

**Bacterial strain**	**Sodium arsenate concentration (mg kg^**−1**^ soil)**	**CFU g**^****−1****^ **of Alfalfa**		**CFU g**^****−1****^ **of Chickpea**	
		**Fresh shoot**	**Fresh root**	**Fresh shoot**	**Fresh root**
*Acinetobacter* sp. QNA2	0	5.6 × 10^3^	1.1 × 10^4^	–	–
	10	5.2 × 10^3^	5.3 × 10^3^	-	–
	50	1.8 × 10^3^	3.2 × 10^3^	–	–
	75	1.5 × 10^3^	3.7 × 10^3^	–	–
	100	6.6 × 10^2^	1.6 × 10^3^	–	–
*Pseudomonas* sp. QNC1	0	–	–	6.5 × 10^3^	1.3 × 10^4^
	10	–	–	4.3 × 10^3^	7.3 × 10^3^
	50	–	—-	4.5 × 10^3^	4.5 × 10^3^
	75	–	–	3.0 × 10^3^	4.0 × 10^3^
	100	–	–	2.0 × 10^3^	2.2 × 10^3^
*Rahnella* sp. QNC2	0	–	–	5.5 × 10^3^	8.7 × 10^3^
	10	–	–	4.7 × 10^3^	5.3 × 10^3^
	50	–	–	2.5 × 10^3^	4.2 × 10^3^
	75	–	–	1.2 × 10^3^	2.8 × 10^3^
	100	–	–	3.3 × 10^2^	2.5 × 10^3^

### Gene Expression

**Figure 6:**
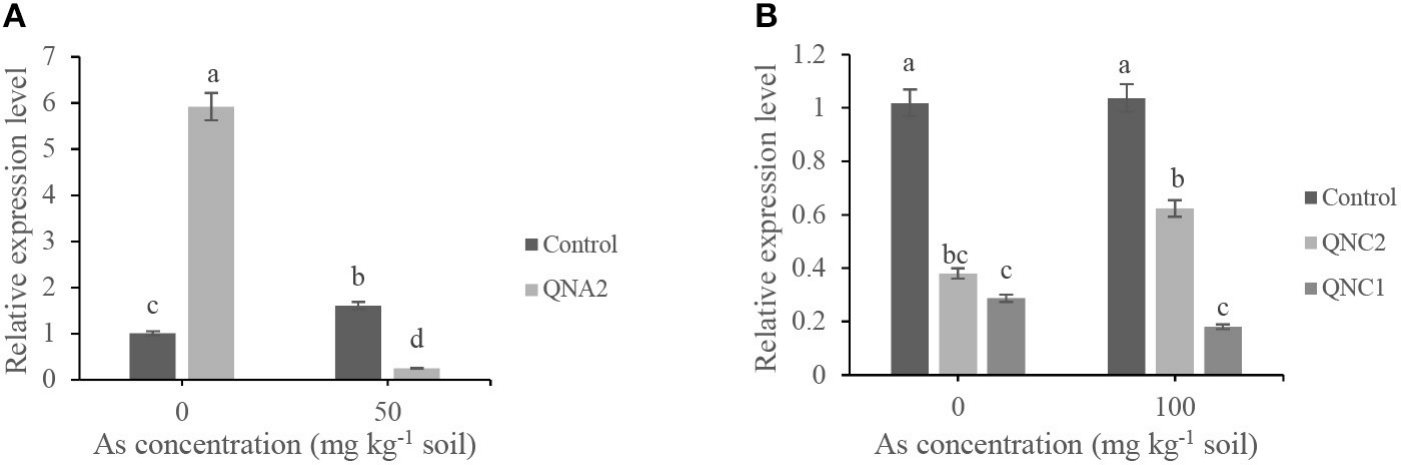
Relative expression levels of the *NPR3* gene in the roots of **(A)** non-treated alfalfa plants (control) and plants treated by *Acinetobacter* sp. QNA2, **(B)** non-treated chickpea plants (control), and plants treated by *Pseudomonas* sp. QNC1 and *Rahnella* sp. QNC2. The results represent the means of three replicates. Vertical bars indicate standard errors (SE), and different letters indicate statistically significant differences between treatments at probability levels of 5%.

qRT-PCR was conducted to examine the relative expression levels of the *NPR3* gene in roots of alfalfa plants treated by QNA2 or treated-chickpea plants by QNC1 and QNC2. The control treatment (plants grown in soil without As) was defined as calibrator and is expressed in the graphs with the value 1, and all the treatments were compared with the control. The results revealed that the expression of the *NPR3* gene in the roots of alfalfa plants treated with QNA2 grown in soil without sodium arsenate was 5.5-fold higher than the control. Surprisingly, the expression level of the *NPR3* gene in QNA2-treated alfalfa plants was 10-fold lower than control when the soil was supplemented with 50-mg/kg of sodium arsenate (Figure 6A). Significant differences were observed between the expression level of the *NPR3* gene in the roots of chickpea plants treated by QNC1 and QNC2 strains compared with the control (Figure 6B). The relative expression level of the *NPR3* gene was significantly decreased in chickpea plants treated by both strains in soil without or with 100 mg kg^−1^ of sodium arsenate compared with the control.

## Discussion

Arsenic is one of the most hazardous environmental pollutants worldwide. It is present in high concentrations in some areas of Kurdistan province and has direct effects on human health. Although both organic arsenic and inorganic arsenic are toxic to organisms, some microorganisms are resistant to this heavy metal due to its genetic makeup. It is well-documented that endophytic bacteria can confer heavy metal resistance to plants due to their metal resistance capacity (Ma et al., 2016). Previous reports indicated that bacteria resistant to arsenic mostly belong to phyla *Proteobacteria, firmicutes*, and *Actinobacteria* (Sheik et al., 2012). Several genera like *Bacillus, Paenibacillus, Staphylococcus, Kocuria, Sphigomonas, Arthrobacter, Brevibacterium, Pseudomonas, Microbacterium, Enterobacter, Stenotrophomonas*, and *Rhizobium* have been reported as arsenic-resistant endophytic bacteria (Zhu et al., 2014; Román-Ponce et al., 2016, 2018; Tiwari et al., 2016).

Our finding in the present study revealed the characterization of endophytic bacteria belonged to the genera *Pseudomonas, Acinetobacter*, and *Rahnella*. *Pseudomonas* is one of the most versatile bacteria with a dynamic distribution of species that adapted to different ecological environments. *Pseudomonas* species were previously reported as As-resistant endophytic bacteria in contaminated areas (Gu et al., 2018). Moreover, the existence of species from *Acinetobacter* and *Rahnella* genera as endophytic bacteria with plant growth promotion activity has also been reported by many researchers (He et al., 2013; Suzuki et al., 2014). All identified strains could grow in media, containing a high concentration of As, which displayed the minimum inhibitory concentration of 300–350 mM for sodium arsenate under our experimental conditions. The ability of bacterial cells to tolerate high concentrations of As contributes to their ecological fitness. This finding is in agreement with previously reported the As-resistant properties of bacterial strains belonging to the same genera (Anderson and Cook, 2004; Valverde et al., 2011; Marwa et al., 2018). Our results suggest that adaptation of such endophytic bacteria to contaminated sites is a result of long-term exposure to high As concentrations (Cai et al., 2009). The resistance property was confirmed by PCR, using primers derived from the *arsC* gene. The *arsC* gene encodes a cytoplasmic arsenate reductase and is highly conserved among microorganisms (Mukhopadhyay et al., 2002). This gene has an essential function in the arsenic resistance mechanism. The presence of the *arsC* gene in strains identified in this study confirms the resistance to arsenic based on the *ars* operon mechanism. In a previous study, As-resistant rhizospheric bacteria were also isolated and characterized from plants (Saadati et al., 2017). The results presented in this study revealed that the resistance of endophytic bacterial strains to As is moderately lower than that of rhizospheric bacteria. It is well-known that, compared with endophytic bacteria, rhizospheric bacteria are more resistant to environmental stress because they are in direct contact with the outside environment (Abedinzadeh et al., 2019).

All endophytic bacteria strains reported in the current study were able to colonize and/or spread throughout the internal tissues of plant hosts. Plant colonization is an essential factor in the efficacy of bacteria to further stimulate plant growth (Silva et al., 2003). All strains exhibited at least one hydrolytic enzyme activity, thus demonstrating that such enzymatic activities may aid in the colonization of the plant hosts (Wang and Dai, 2010). Endophytic bacteria have several competitive advantages over other bacteria, because of their close and continued contact with plants. There is increasing interest in finding beneficial endophytic bacteria and modulating their potential application for improving plant growth under stress conditions. Bacterial strains characterized in this study showed multiple plant growth promotion traits. This indicates that As contamination has not been able to prevent the production of such properties.

There are many endophytic bacteria reported to be directly or indirectly involved in plant growth promotion by siderophore production, nitrogen fixation, and solubilization of phosphate (Compant et al., 2010). Siderophores are organic molecules with a high affinity for Fe (III), thus participating in iron availability for plants, even under limited essential metal availability or in polluted soil (Rajkumar et al., 2010, 2012; Schalk et al., 2011). The previous report indicated that siderophore forms a stable complex with As; thus, the application of bacteria with siderophore production activity proves beneficial for As remediation (Jeong et al., 2014). Several reports have indicated that different endophytic bacterial species can solubilize insoluble inorganic phosphate compounds. These bacteria make available the soluble phosphates to the plant hosts (Oteino et al., 2015). In addition, arsenic is an analog of phosphorus that enters through phosphate transporters into the roots (Pinter et al., 2017). In addition to As-resistance, detection of some of the plant growth promotion properties among the strains in the present study indicates that these endophytic bacteria may help their plant host to resist in the arsenic-contaminated areas, although their ability to express such properties under high As concentration needs to be investigated. Because these bacteria were isolated from arsenic-contaminated sites, they have the advantage of being adapted to such stressful situations.

In the present study, the high As concentration added to the soil negatively affected plant growth. Usually, a high concentration of As in the soil interferes with the normal absorption of nutrients like P and Fe, which results in inhibiting plant growth. In this context, inoculation with endophytic bacteria showed an improved positive effect on the number of nodules, shoot and root biomass compared with the non-treated plants. This effect can occur due to their ability to solubilize phosphate, siderophore production, and/or fix nitrogen to provide a suitable form of P, Fe, and N, resulting in stimulating plant growth. It is well-documented that many non-rhizobial bacteria are nitrogen fixers and induce nitrogen-fixing nodules on legume roots (Martinez-Hidalgo and Hirsch, 2017). Results presented in this study indicated that inoculation of plants with endophytic bacteria showed increasing effects on the number of nodules. Although confirming the direct role of these bacteria in inducing nodule formation needs more experiments. Based on the results obtained, all bacterial strains tested could colonize roots and shoots of inoculated alfalfa and chickpea plants grown in soil supplemented with different concentrations of As. It was previously reported that the ability of bacteria to colonize plant tissues made them more effective for enhancing beneficial effects (Mallick et al., 2014).

Induction of systemic resistance is the immunity response mechanism in plants that can be triggered by endophytic bacteria during abiotic stress conditions, including heavy metal (Ghosh et al., 2020). The relative expression level of the *NPR3* gene in alfalfa and chickpea plants treated with endophytic bacteria in soil supplemented with 50- and 100-mg kg^−1^ concentrations of As, respectively, was analyzed. As shown in Figure 4, in most experiments, higher significant differences were obtained between QNA2-treated alfalfa plants and non-treated control under 50 mg kg^−1^ of sodium arsenate. Similarly, based on the results presented in Figure 5, in most experiments, higher significant differences were obtained between QNC1, QNC2-treated chickpea plants, and non-treated control under 100 mg kg^−1^ of sodium arsenate. Therefore, we have determined the relative expression levels of the *NPR3* gene in alfalfa and chickpea plants grown under 50 and 100 mg kg^−1^ of sodium arsenate, respectively. Before RT-PCR, analysis of primers specificity was conducted by the heat dissociation test. The corresponding dissociation curve shows primers specificity, and the heat dissociation curve was formed at 60°C. It is well-documented that salicylic acid (SA) ameliorates As toxicity in plants. Salicylic acid is reported to eventually trigger the NPR1 (Non-expresser of Pathogenesis-Related genes1) mediated pathway for systemic acquired resistance (SAR) in plants under stress (Klessig et al., 2000). In addition, the NPR1 paralogues NPR3 and NPR4 are SA receptors that mediate NPR1 degradation in an SA-regulated manner (Fu et al., 2012). The results presented in this study indicated that the expression level of the *NPR3* gene in the roots of alfalfa and chickpea plants induced a low level or remained unchanged, respectively, when As was added to the final concentration of 50 and 100 mg kg^−1^ of soil. These results suggest that As cannot induce systemic-acquired resistance *via* an NPR-dependent pathway. In contrast, upon an endophytic bacteria challenge, treated plants demonstrated a lower abundance of *NPR3* gene expression compared with the non-treated plants, which indicates activated NPR-dependent defense pathways.

In conclusion, we isolated and characterized several arsenic-resistant endophytic bacteria from the root of leguminous plants growing in arsenic-contaminated areas in Kurdistan province, Iran for the first time. All bacterial strains showed a high level of tolerance to As. This finding suggests that the acquisition of tolerance to As may play an essential role in an adaptation of bacteria to As. All these bacteria studied had a beneficial effect on the plants and promoted growth in soil supplemented with As. The As-resistance and plant growth promotion properties of these bacteria demonstrate their potentiality for the establishment of plants in As-contaminated environments. Further studies will be done on understanding the mechanisms involved in the interactions between plant hosts and such endophytic bacteria finding applications as a bioremediation tool with great ecological importance.

## Data Availability Statement

The datasets presented in this study can be found in online repositories. The names of the repository/repositories and accession number(s) can be found in the article.

## Author Contributions

HT conducted all the experiments and analyzed all the data but was assisted by JR, BH, and AA performed the gene expression quantification experiments. BH wrote the manuscript. All the authors read and approved the manuscript.

## Conflict of Interest

The authors declare that the research was conducted in the absence of any commercial or financial relationships that could be construed as a potential conflict of interest.
